# Chronic kidney disease and its associated factors in HIV-infected individuals: a comparison of antiretroviral therapy naïve and experienced patients

**DOI:** 10.3389/fmed.2024.1455688

**Published:** 2024-11-11

**Authors:** Daniel Asmelash, Marye Nigatie

**Affiliations:** ^1^Department of Medical Laboratory Science, College of Medicine and Health Sciences, Mizan-Tepi University, Mizan-Aman, Ethiopia; ^2^Department of Medical Laboratory Sciences, College of Health Sciences, Woldia University, Woldia, Ethiopia

**Keywords:** chronic kidney disease, associated factors, prevalence, highly active antiretroviral therapy, naïve, experienced, HIV

## Abstract

**Background:**

Chronic kidney disease (CKD) has emerged as one of the primary comorbidity affecting individuals infected with human immunodeficiency virus (HIV), even after the initiation of highly active antiretroviral therapy (HAART). The main objective of this study was to assess the prevalence of CKD and its associated factors among HIV-infected individuals who are HAART naïve compared to those who are HAART experienced.

**Methods:**

An institution-based cross-sectional study was conducted at Mizan Tepi University Comprehensive Specialized Hospital from March to May 2022. A double population proportion formula was used to select 250 study participants, with 125 being HAART naïve and 125 being HAART experienced. Socio-demographic and clinical data were collected using a semi-structured questionnaire. Serum creatinine levels were measured using a Mindray BS-200 chemistry analyzer, and the estimated glomerular filtration rate (eGFR) was calculated using the Modification of Diet in Renal Disease (MDRD) equation. The level of urine protein was measured using a reagent strip within 30 min of collection. Descriptive statistics, independent t-tests, and multivariable logistic regression analysis were performed, with a *p*-value of <0.05 considered statistically significant.

**Result:**

The mean (±SD) age of the HAART-naïve individuals was 35 ± 9.5, while that of the HAART-experienced individuals was 45 ± 9.9 years. Of the total participants, 67.2% participants were women. The overall prevalence of CKD among the HIV-infected study participants was 36.4%. The prevalence of CKD was 33.6% in HAART-naïve individuals and 39.2% in HAART-experienced individuals, with a *p*-value of 0.03. Male sex was identified as an independent factor associated with CKD in this study.

**Conclusion:**

The prevalence of CKD was found to be higher among HAART-experienced individuals than HAART-naïve individuals. Regular renal function assessments should be conducted before and during HAART to mitigate the risk of renal dysfunction.

## Introduction

The human immunodeficiency virus (HIV) is a retrovirus that targets immune system cells, destroying or damaging their function (1). Since the global treatment initiative began in 2000, annual AIDS-related deaths have significantly declined. According to estimates from 2023, approximately 39.9 million people are living with HIV/AIDS, and 630,000 people died from AIDS-related diseases.

Since 2020, AIDS-related deaths have reduced by 56% among women and girls and by 47% among men and boys. In sub-Saharan Africa, new HIV infections are particularly high, with women and young girls accounting for 62% of these cases. In contrast, the percentage difference between estimates in other geographical regions is relatively small, suggesting that the coverage of births in the dataset is realistic.

As of December 2023, there were 30.7 million people on antiretroviral therapy (ART), an increase from 7.7 million in 2010, with a target of 34 million by 2025 (2). Eastern and Southern Africa remain the most affected regions, with approximately 20.6 million people living with HIV in Eastern Africa alone in 2022. Overall, sub-Saharan Africa accounts for 76% of all HIV-infected individuals, 76% of all new HIV infections, and 75% of all HIV/AIDS-related deaths globally (2, 3).

A total of 610,350 people are living with HIV, 8257 new HIV infections, and 11,322 annual AIDS-related deaths, according to estimates from the Ethiopian Public Health Institute (EPHI) for 2022 and 2023. National estimates for PLHIV, new HIV infections, and AIDS-related deaths in 2022 and 2023 show a reduction of 1, 12.8, and 11.8%, respectively (4).

The introduction of highly active antiretroviral therapy has led to a noticeable reduction in HIV-related mortality (5). However, new health complications, including chronic kidney disease (CKD), have emerged. The increase in life expectancy following the introduction of HAART and the long-term development of metabolic complications such as diabetes and dyslipidemia, hypertension, and vascular diseases can contribute to the increasing frequency in the recognition of renal impairment in HIV-infected patients (6).

CKD has become a prominent comorbidity in people living with HIV, especially those on long-term ART with protease inhibitors (7). Renal disease in HIV-infected individuals is a contributing factor to morbidity and mortality (8).

The pathology of CKD is complex and includes causes such as direct viral toxicity, immune activation, and ART-induced nephrotoxicity. Studies have shown that HIVAN is mediated by viral gene expression, particularly in the podocytes, and the role of genetic predisposition is significant, with APOL1 gene variants being a key risk factor for CKD progression in African populations (4, 9). Studies show that risk factors associated with CKD in HIV-infected populations include aging, female sex, hypertension, diabetes mellitus, low CD4 cell count, and ART exposure (10–12).

Renal disorders in HIV patients are noticeable, ranging from fluid and electrolyte imbalances to end-stage renal disease (ESRD) regardless of HIV stages. Treatment-related factors, recurrent viremia, and traditional risk factors may be attributable to CKD (13). Renal function impairment that results from HIV infection is called human immunodeficiency virus-associated nephropathy (HIVAN), which is predominantly prevalent in black people, especially in the sub-Saharan population (14, 15). HIVAN is mediated by factors related to the virus, host genetic predisposition, and environmental factors. The host response to HIV infection may influence disease phenotype by activating cytokine pathways (16).

Antiretroviral (ARV) drugs can also result in renal damage, which is presenting as acute kidney injury (AKI), tubular necrosis, kidney stones, or CKD (14, 17). The mechanism by which ART causes renal toxicity seems to be related to drug accumulation within proximal renal tubules, leading to mitochondrial DNA injury, depletion, and dysfunction of the oxidative respiratory chain in proximal tubular epithelial cells. This may lead to a depletion of intracellular adenosine triphosphate, which limits the proximal tubule’s ability to reabsorb electrolytes and low-molecular-weight proteins (18–20).

Renal toxicity associated with the use of nucleoside analogs is generally rare. However, case reports have demonstrated that treatments involving didanosine and lamivudine-stavudine can lead to tubular dysfunction (21, 22). Tenofovir and cidofovir, both acyclic nucleoside phosphonates, are also associated with renal tubular damage. The adverse renal effects may present in a variety of clinical forms, ranging from tubule cell death, such as acute tubular necrosis, to possibly reversible tubular dysfunction (23). HAART regimens containing the combination of tenofovir/atazanavir are associated with a low glomerular filtration rate (eGFR), which increases the risk of CKD development (24). Generally, the renal system excretes many drugs and their metabolites through the proximal tubule. Consequently, drug-related damage can occur due to the high blood flow and elevated toxin levels present in the proximal tubule (18).

The purpose of this study was to assess CKD among HIV-infected patients attending the Mizan Tepi Hospital. In addition, this study will give reliable information regarding associated factors that may increase the development of CKD. The findings will be supportive of early diagnosis and treatment of HIV-related renal dysfunction. It will also be helpful for clinicians to know the associated factors in managing HIV/ADIS patients.

## Methods and materials

### Study area

The study was conducted at the outpatient HIV clinic of Mizan Tepi Hospital, Ethiopia. The hospital is one of the biggest hospitals in southwest Ethiopia, providing health services and acting as the referral center for other district hospitals. The ART clinic gives service to a total of over 2,463 HIV-infected individuals.

### Study design

A hospital-based comparative cross-sectional study was conducted to compare the CKD and associated factors between HAART-naïve and HAART-experienced HIV-infected individuals from March to May 2022.

### Inclusion and exclusion criteria

All individuals aged ≥18 years old with HIV were included in the study, comprising both HAART-naïve and HAART-experienced individuals. HAART-naïve individuals were those who had never received antiretroviral therapy, while HAART-experienced participants were selected after one year of antiretroviral treatment. Severely ill patients unable to communicate, hospitalized patients, pregnant women over 65 years of age, and amputees were excluded from the study.

### Definitions

HAART-experienced persons who took HAART for more than 1 year, which is composed of two NRTIs plus an NNRTI (25). CKD is defined as kidney damage or glomerular filtration rate (GFR) <60 mL/min/1.73 m^2^ for 3 months or more, irrespective of cause (26). The eGFR was estimated using the Modification of Diet in Renal Disease (MDRD) Study equation: 186 × ${\left[\text{serum creatinine}\;\left(\frac{\text{mg}}{\text{dl}}\right)\right]}^{-1.154}$× ${\left(\text{age}\right)}^{-0.203}$ × (0.742 if female) × (1.210) (3). CKD is classified based on cause, GFR category, and albuminuria category (27).

- Stage 1, persistent proteinuria with eGFR ≥90 mL/min/1.73 m^2^,
- Stage 2, persistent proteinuria with eGFR of 60–89.9 mL/min/1.73 m^2^,
- Stage 3, eGFR 30–59.9 mL/min/1.73 m^2^ with or without proteinuria,
- Stage 4, eGFR 15–29.9 mL/min/1.73 m^2^ with or without proteinuria, and
- Stage 5 (kidney failure), eGFR<15 mL/min/1.73 m^2^ with or without proteinuria.

### Sample size

The sample size was calculated using a double population proportion formula, based on two different proportions of chronic renal disease: 3.6% for HAART-naïve participants and 11.7% for HAART-experienced participants, derived from a previous study in Northwest Ethiopia (28). A critical value was set at a 95% confidence level, with a significance level of 0.05 and a power (1−*β*) of 90. A 10% compensation was added to account for potential non-responses, resulting in a total sample size of 250 individuals (125 HAART-naïve and 125 HAART-experienced individuals).


$$N=2\times \left(p\right)\left(1-p\right)\left(z\beta +\frac{z\propto }{2}\right)2/\left(p1-p2\right)2$$

$$=2\left(0.0765\right)\;\left(1–0.0765\right)\;{\left(1.28\times 1.96\right)}^{2}/{\left(0.036–0.117\right)}^{2}$$

=2×(0.0765)×(0.9235)×(10.4976)/0.006561

=226.0728≈227

=227×10%,Assuming10%non−response rate.

=22.7≈23,=227+23=250

where *n* = minimum sample size, p = best estimate of the double population proportion, p=p1+p2⋰2, *p*1 = proportion of CKD for HAART naïve = 3.6%, *p*2 = proportion of CKD for HAART-experienced = 11.7%, Z*_β_* = Power (1−*β*) =90% = 1.28, *Z*_α/2_ = level of significance = 0.05.

### Data collection and laboratory analysis

Socio-demographic characteristics and clinical data were collected by trained nurses using a semi-structured questionnaire. In addition, qualified personnel took anthropometric and blood pressure measurements. Five milliliters of venous blood samples were collected using serum separator test tubes. The specimen was left at room temperature for a minimum of 30 min to clot and then centrifuged at 300 RPM for five minutes. A serum creatinine level was measured using a Mindray BS-200 chemistry analyzer (Shenzhen Mindray Bio-Medical Electronics Co. Ltd., China). An estimated glomerular filtration rate (eGFR) was calculated using the Modification of Diet in Renal Disease (MDRD) equation. Fifty mL of freshly voided urine were collected in sterile containers. According to manufacturer instructions, proteinuria was assessed using a reagent strip within 30 min after collection.

### Statistical analysis

Data were entered into Epi Info version 3.5.1 and then transferred to SPSS version 20 for analysis. The prevalence of CKD was compared between HAART-naïve and HAART-experienced individuals using independent t-tests for continuous variables and chi-square tests for categorical variables. Bivariable and multivariable logistic regression analyses were performed to identify CKD-associated factors. The multivariable model used a backward, stepwise elimination approach, retaining variables with a *p*-value of <0.25 from the bivariable analysis. This method involves initially including all candidate variables in the model and then sequentially removing those that do not significantly contribute to the model based on the likelihood ratio test or pre-specified criteria (such as *p*-values). The least significant variable is removed in each step, and the process is repeated until only statistically significant predictors remain. A p-value <0.05 was considered statistically significant.

## Results

### Socio-demographic characteristics of study participants

From a total of 250 HIV-infected study participants, 168 (67.2%) were women. The mean (±SD) age of the study participants was 35 ± 10 and 45 ± 10 years for HAART naïve and experienced patients, respectively. The majority (85.6%) of the participants were urban inhabitants. In this study, 47 (18.8%) and 28 (11.2%) of study participants were alcohol drinkers and cigarette smokers, respectively (Table 1).

**Table 1:** Socio-demographic characteristics of HIV-infected participants, 2022 (*N* = 250).

Variables		HAART naive	HAART-experienced	Total
		No (%)	No (%)	No (%)
Age (years)	18–28	37(29.6)	1(0.8)	38(15.2)
	29–38	47(37.6)	36(28.8)	83(33.2)
	39–48	29(23.2)	46(36.8)	75(30.0)
	≥40	12(9.6)	42(33.6)	54(21.6)
Sex	Male	47(37.6)	35(28.0)	82(32.8)
	Female	78(62.4)	90(72.0)	168(67.2)
Residency	Urban	106(84.8)	108(86.4)	214(85.6)
	Rural	19(15.2)	17(13.6)	36(14.4)
Marital status	Single	15(12.0)	11(8.8)	26(10.4)
	Married	73(58.4)	68(54.4)	141(56.4)
	Widowed	11(8.8)	31(24.8)	42(16.8)
	Divorced	26(20.8)	15(12.0)	41(16.4)
Educational level	Unable to read and write	30(24.0)	40(32.0)	70(28)
	Primary	47(37.6)	37(29.6)	84(33.6)
	Secondary	32(25.6)	31(24.8)	63(25.2)
	Higher	16(12.8)	17(13.6)	33(13.2)
Occupation	Unemployed	53(42.4)	39(31.2)	92(36.8)
	Self-employed	50(40.0)	58(46.4)	108(43.2)
	Governmental	22(17.6)	28(22.4)	50(20)
Smoking habit	No	107(85.6)	115(92.0)	222(88.8)
	Yes	18(14.4)	10(8.0)	28(11.2)
Alcohol habit	No	81(64.8)	122(97.6)	203(81.2)
	Yes	44(35.2)	3(2.4)	47(18.8)

HAART, Highly Active Anti-Retroviral Therapy; HIV, Human Immunodeficiency Virus.

### Clinically related information

Approximately 55 (22%) of total HIV-infected study participants had stage II CKD. In addition, less than half, 10 (8.0%) of HAART-naïve and 12 (9.6%) of HAART-experienced study participants had stage III CKD, respectively. Approximately all 123 (98.4%) HAART-experienced study participants received first-line ART regimens, and more than half (54.4%) of HAART-experienced study participants were non-TDF regimen users. The majority of 118 (94.4%) HAART-naïve study participants were < 5 years since diagnosis of HIV. On the other hand, over half, 77 (61.6%) of HAART-experienced participants were above 5 years after being diagnosed. History of hypertension and diabetes mellitus were reported in 12 (4.8%) and 11 (4.4%) of the participants, respectively (Table 2).

**Table 2:** HIV-related information among HIV-infected participants, 2022 (*N* = 250).

Variables		HAART naïve	HAART experienced	Total
		No (%)	No (%)	No (%)
BMI (kg/m^2^)	<18.5	37(29.6)	19(15.2)	56(22.4)
	18.5–24.9	61(48.8)	78(62.4)	139(55.6)
	25–29.9	25(20.0)	23(18.4)	48(19.2)
	≥30	2(1.6)	0(0)	7(2.8)
Time since diagnosis of HIV (years)	<5	125(100)	40(32.0)	158(63.2)
	5–10	0(0)	66(52.8)	73(29.2)
	>10	0(0)	19(15.2)	19(7.6)
WHO HIV stage	I	44(35.2)	17(13.6)	30(12)
	II	57(45.6)	84(67.2)	128(51.2)
	III	13(10.4)	23(18.4)	80(32)
	IV	10(8.0)	1(0.8)	12(4.8)
CD4+ (cells/mm^3^)	<200	58(46.4)	28(22.4)	86(34.4)
	200–350	42(33.6)	38(30.4)	80(32)
	>350	25(20.0)	59(47.2)	84(33.6)
Time since on ART (years)	<5		48(38.4)	
	5–10		35(28.0)	
	>10		42(33.6)	
ART regimen	First		123(98.4)	
	Second		2(1.6)	
Diabetes mellitus history	No	117(93.6)	122(97.6)	239(95.6)
	Yes	8(6.4)	3(2.4)	11(4.4)
Hypertension history		120(96)	118(94.4)	238(95.2)
		5(4)	7(5.6)	12(4.8)
Acute kidney injury	No	99(79.2)	97(77.6)	196(78.4)
	Yes	26(20.8)	28(22.4)	54(21.6)
Kidney stone history	No	114(91.2)	118(94.4)	232(92.8)
	Yes	11(8.8)	7(5.6)	18(7.2)
First-line regimen types	1c		47(37.6)	
	1d		20(16.8)	
	1e		48(38.4)	
	1f		5(6.4)	

1c = AZT + 3TC + NVP, 1d = AZT + 3TC + EFV, 1e = TDF + 3TC + EFV, 1f = TDF + 3TC+ NVP = 3TC = lamivudine; AZT, zidovudine; NVP, nevirapine; EFV, efavirenz; TDF, Tenofovir Disoproxil Fumarate; CD4, Cluster of Differentiation4; WHO, World Health Organization; HIV, Human Immunodeficiency Virus; ART, Anti-Retroviral Therapy; HAART, Highly Active Anti-Retroviral Therapy.

### Renal function tests in HAART-naïve vs. HAART-experienced patients

The mean (±SD) of Cr level was 1.1 ± 0.7 mg/dL for HAART naïve and 0.98 ± 0.2 mg/dL for those who were HAART-experienced, with *p* = 0.01. The mean (±SD) of CrCl in HAART naive and HAART-experienced study participants were 73.8 ± 33.2 and 67.8 ± 19.2, respectively (*p* = 0.08). In urine protein testing, 33 (26.4%) of HAART-naïve patients showed proteinuria of 1+ or higher. However, 43 (34.4%) of HAART-experienced had proteinuria 1+ and above. The mean (±SD) of eGFR (MDRD formula) of HAART naïve and experienced were 96.0 ± 44.8 mL/min/1.73 m^2^ and 85.3 ± 20 mL/min/1.73 m^2^, respectively. HAART-naïve study participants had a significantly higher mean eGFR than HAART-experienced participants (*p* = 0.01).

### Prevalence of CKD among HIV-infected study participants

The prevalence of CKD among HIV-infected participants was 36.4% (121). The prevalence of CKD among HAART-naïve study participants was 33.6%, whereas among HAART-experienced study participants was 39.2%. The majority, 22%, of CKD cases were observed in CKD stage 2 among total HIV-infected study participants (Table 3).

**Table 3:** Prevalence of CKD based on MDRD formula and proteinuria among HIV-infected participants, 2022 (*N* = 250).

Variables			HAART naïve	HAART-experienced	Total
			No (%)	No (%)	No (%)
CKD		No	83(66.4)	76(60.8)	159(63.6)
		Yes	42(33.6)	49(39.2)	91(36.4)
Stages	1	≥ 90	6(4.8)	7(5.6)	13(5.2)
	2	60–89	25(20)	30(24)	55(22)
	3	30–59.9	10(8.0)	12(9.6)	22(8.8)
	4	15–29	0(0)	0(0)	0(0)
	5	< 15	1(0.8)	0(0)	1(0.4)
Proteinuria	No		92(73.6)	82(65.6)	174(69.6)
	Yes		33(26.4)	43(34.4)	102(30.4)

Proteinuria ≥ 1+; CKD, Chronic Kidney Disease; MDRD, Modification of Diet and Renal Disease; HAART, Highly Active Anti-Retroviral Therapy; HIV, Human Immunodeficiency Virus.

### Factors associated with CKD among HIV-infected study participants

The association of CKD between HAART-naïve and HAART-experienced study participants was statistically significant (Table 4). Multivariable logistic regression analysis showed that male gender was significantly associated with CKD among HIV-infected study participants (*p*-value =0.03) (Table 5).

**Table 4:** Mean of biochemical parameters and association of biochemical between HAART naive and HAART-experienced individuals, 2022 (*N* = 250).

Parameters		HAART naïve	HAART-experienced	Total	*p* value
		Mean ± SD	Mean ± SD	Mean ± SD	
Cr (mg/dL)^a^		1.1 ± 0.7	0.98 ± 0.2	1.04 ± 0.45	0.01*
CrCl C-G (mL/min)		73.8 ± 33.2	67.8 ± 19.2	70.8 ± 26.2	0.08
eGFR (MDRD) (mL/min/1.73m^2^)		96.0 ± 44.8	85.3 ± 20.0	90.65 ± 32.4	0.01*
CD4 (cells/mm^3^)		245.9 ± 163.5	391.3 ± 209.5	318.6 ± 186.5	0.01*
Proteinuria	Neg.	92(73.6)	82(65.6)	174(69.6)	0.378
	1+ and above	33(26.4)	43(34.4)	102(30.4)	
CKD	No	83(66.4)	76(60.8)	159(63.6)	0.03*
	Yes	42(33.6)	49(39.2)	91(36.4)	

a Based on single measurement.

*Significant; Cr, Creatinine; CrCl, Creatinine Clearance; eGFR, estimated Glomerular Filtration Rate; MDRD, Modification of Diet and Renal Disease; CD4, Cluster of Differentiation 4; HAART, Highly Active Anti-Retroviral Therapy.

**Table 5:** Factors associated with CKD among HIV-infected participants 2022 (*N* = 250).

Factors		CKD	CKD	COR(95%CI)	AOR(95%CI)	*p* value
		Yes	No			
Gender	Men	30	52	1.5(0.9–2.5)	2.7(1.1–6.6)	0.03*
	Women	61	107	1		
Age group	≤40					
	≥40					
CKD family history	No	84	130	1		
	Yes	7	29	0.5(0.2–1.3)	0.5(0.1–2.0)	0.30
CD4 (cells/mm^3^)	<200	33	53	1.6(0.9–2.7)	2.5(0.9–6.6)	0.07
	≥200	58	106	1		
HIV duration (yr.)	<6	61	114	1		
	≥6	30	45	1.9(1.1–3.4)	1.3(0.6–2.9)	0.50

*Significant; TDF, Tenofovir Disoproxil Fumarate; CD4, Cluster of Differentiation 4; yr, year; COR, Crude Odds Ratio; AOR, adjusted odds ratio, backward stepwise multiple logistic regression was used to assess the independent effect of explanatory variables.

## Discussion

Renal disease has been documented as a common and closely associated complication of HIV infection (29). HIV-infected individuals can have many adverse factors that can affect the kidneys and arise from direct effects of HIV, such as immune complex disease and HIVAN, or indirectly from opportunistic infection and drugs (30).

The prevalence of CKD among HIV-infected study participants in this study, based on eGFR calculated using the MDRD formula, was 36.4%. The current study result was higher than studies conducted in China (16.8%) (10), northwest Ethiopia (21.5%) (31), Belgium (3%) (32), Brazil (8.4%) (33), and sub-Saharan Africa (7%) (34). The observed difference may be due to geographical variation, study design, and sample size.

The prevalence of CKD among HAART-naïve study participants was 33.6%, lower than in studies conducted in Nigeria (53.3%) (14) but much higher than in studies from China (16.1%) (35), Kenya (11.5%) (36) and Malawi (21%) (37). This variation might be due to differences in geographical distribution and sample size.

On the other hand, the prevalence of CKD among HAART-experienced study participants in this study (39.2%) was higher as compared to studies conducted in Ghana (9.9%) (38), Nigeria (23.8%) (39), and Tanzania (25%) (40). Differences in GFR estimation methods, such as the use of the C-G formula in Nigeria and Tanzania, as well as the retrospective design and sample size of the Nigerian study, may contribute to the observed differences. The present study also observed a higher prevalence of CKD in the HAART-experienced group than HAART-naïve. Cross-sectional studies demonstrate a higher estimated prevalence of CKD in HAART-experienced persons than in HAART-naïve individuals, pointing to the adverse effects of certain antiretroviral drugs and the overall duration of HIV exposure (33, 41). The prevalence of CKD in HIV-infected patients also differs in different regions of the world. CKD prevalence in Africa is between 6 and 48.5% (42). In our study, although we did not specifically differentiate HIVAN, the high prevalence of proteinuria in HAART-naïve individuals could suggest early-stage kidney damage, consistent with reports from other low-resource settings.

In this study, the observed difference in CKD prevalence between HARRT naïve and experienced study participants was statistically significant (*p* = 0.03). This is consistent with a study conducted in Burundi (43) and Northwest Ethiopia (44). The reason might be due to the type of drug used, age, sample size, and duration of HAART. The higher prevalence of CKD among HAART-experienced individuals may be due to different factors, including HIV infection duration, medication adherence, comorbidities, and different drugs. Therefore, to avoid these risks, clinicians should closely monitor the renal function of HIV-infected patients on HAART and those at increased risk for the development of CKD (41).

In this study, the prevalence of proteinuria was 30.4, 26.4, and 34.4% among total study participants, HAART-naïve and HAART-experienced participants, respectively. This study demonstrates high proteinuria when compared with studies conducted in China (13.7%) (35), India (21%) (45), Malawi (23.3%) (37), Burundi (6.1%) (43), Kenya (12%) (30), and northwest Ethiopia (Bahir Dar) (17.9%) (31). On the other hand, a higher finding was observed in Nigeria (41.4%) (46). Sample size, geographical variation, and the use of different measuring techniques for proteinuria may contribute to the observed difference. The high prevalence of proteinuria observed in the present study, particularly among HAART-experienced patients, has important clinical implications for CKD management in HIV-positive individuals. In HIV-infected individuals, proteinuria may indicate HIV-associated nephropathy, possibly due to ART nephrotoxic effects or TDF-containing regimens. The presence of proteinuria necessitates regular screening and close monitoring to detect kidney damage at an early stage (47, 48).

The high prevalence of proteinuria observed in the present study and the higher prevalence in HAART-experienced subjects have significant clinical implications for managing CKD in HIV-positive patients. Proteinuria is a standard indicator of renal injury in CKD patients, with increased levels strongly correlating with clinical status and adverse cardiovascular outcomes. Proteinuria in HIV-infected people could be a primary HIV-associated nephropathy indicator due to ART nephrotoxic effects or TDF-containing regimens. This means that if there is proteinuria, there is a need to screen and follow up to ensure that there is no kidney damage yet.

Since renal function typically declines with age, older age is an established risk factor for decreased CrCl (49). According to this study, the observed CKD in overall HIV-infected study participants with age ≥ 40 years were more likely to develop CKD than those <40 years of age. In addition, the result found from this study showed low CD4 count (<200 cells/mm^3^) was significantly associated with CKD among HIV-infected study participants. A low CD4 count (< 200 cells/mm^3^) was 2.7 times more likely to develop CKD among overall HIV-infected study participants. This result is in line with a study conducted in China (10), Nigeria (39), and southwest Ethiopia (44). In this study, the association of CKD with low CD4 cell count may suggest that HIVAN is a contributory cause of CKD in study participants since CD4 cell count is used as a surrogate marker of immunological status in HIV-infected patients (14).

Male gender was independently and significantly associated with CKD in this study (AOR 95% CI, 2.7, 1.1–6.6). This is not in agreement with the findings of studies conducted in France (50), the U.S.A (51), and Burundi (43). However, unlike other similar studies, this study did not find a significant association between CD4 counts, older age, and reduced eGFR (8, 52). The difference could be due to differences in population characteristics, such as lifestyle and cultural behaviors, and risk factors specific to certain regions that may influence the gender association with CKD. The difference in study design could partly explain this difference, as this study used the MDRD equation to estimate the eGFR.

However, in this study, creatinine level and proteinuria were obtained only once; hence, consecutive results could not be compared, which may be a limitation when trying to estimate the presence of CKD since reversal causes may exist. However, since this is a prospective study, comparing HAART status and evaluating factors related to CKD can be regarded as a strength of this study.

## Conclusion

The overall prevalence of CKD and proteinuria found in this study was high. The results indicate that the prevalence of CKD among HAART-experienced study participants was higher than that among HAART-naïve study participants. The current study revealed that the majority of the CKD cases were found in stage 2 of the disease. In addition, the current study revealed that being male is a factor that can predict the likelihood of developing CKD in HIV-infected participants. Regular screening and close monitoring for proteinuria are essential to detect kidney damage at an early stage. Therefore, to minimize these risks, clinicians should closely monitor the renal function of HIV-infected patients on HAART and those at increased risk for developing CKD.

## Data Availability

The raw data supporting the conclusions of this article will be made available by the authors without undue reservation.
